# Positive Allosteric Modulation of Insect Olfactory Receptor Function by ORco Agonists

**DOI:** 10.3389/fncel.2016.00275

**Published:** 2016-12-09

**Authors:** Panagiota Tsitoura, Kostas Iatrou

**Affiliations:** Insect Molecular Genetics and Biotechnology Group, Institute of Biosciences and Applications, National Centre for Scientific Research “Demokritos”Athens, Greece

**Keywords:** *Anopheles gambiae*, mosquito olfaction, ligand discovery, ORco agonists, malaria, olfactory function enhancement, olfactory receptor pharmacology, cell-based screening

## Abstract

Insect olfactory receptors (ORs) are heteromeric ligand-gated cation channels composed of a common olfactory receptor subunit (ORco) and a variable subunit (ORx) of as yet unknown structures and undetermined stoichiometries. In this study, we examined the allosteric modulation exerted on *Anopheles gambiae* heteromeric ORx/ORco olfactory receptors *in vitro* by a specific class of ORco agonists (OAs) comprising ORcoRAM2 and VUAA1. High OA concentrations produced stronger functional responses in cells expressing heteromeric receptor channels relative to cells expressing ORco alone. These OA-induced responses of ORx/ORco channels were also notably much stronger than those obtained upon administration of ORx-specific ligands to the same receptors. Most importantly, small concentrations of OAs were found to act as strong potentiators of ORx/ORco function, increasing dramatically both the efficacy and potency of ORx-specific odorants. These results suggest that insect heteromeric ORs are highly dynamic complexes adopting different conformations that change in a concerted fashion as a result of the interplay between the subunits of the oligomeric assemblies, and that allosteric modulation may constitute an important element in the modulation and fining tuning of olfactory reception function.

## Introduction

Insect odorant receptors (ORs) constitute a family of ligand-gated ion channels (Sato et al., 2008; Wicher et al., 2008) unrelated to the mammalian olfactory receptors, which are members of the G-protein coupled receptor (GPCR) superfamily (Touhara, 2002). They are heteromeric complexes composed of a variable (ORx) and a conserved (ORco) subunit (Nakagawa et al., 2005; Neuhaus et al., 2005; Sato et al., 2008), henceforth ORx/ORco, of as yet unknown stoichiometries. Their study has received much attention both in the context of insect biology and evolution and the potential for pest control applications (Leal, 2010; Carey and Carlson, 2011; Benton, 2015). Despite the differences in olfactory receptor structure and signaling between insects and mammals (Kaupp, 2010; Silbering and Benton, 2010; Getahun et al., 2013), complexity in odor coding applies to insects as much as mammals (Malnic et al., 1999; Hallem and Carlson, 2006).

One of the first insect OR repertoires to be studied due to its potential application for disease vector control (Leal, 2010; Carey and Carlson, 2011), was that of the malaria vector *Anopheles gambiae*, which was initially predicted to consist of 79 ORs [78 ORx members and ORco; (Hill et al., 2002)]. Nearly half of them have been functionally characterized using the *Xenopus* oocyte system (Wang et al., 2010), while up to 50 were functionally expressed in the empty neuron system (Carey et al., 2010). Selectivity and recognition of odorant molecules is determined by the variable subunit (ORx), while ORco is essential for the formation of the channel (Nichols et al., 2011). However, because the structures of insect ORs have yet to be determined, the properties of the odorant binding sites are basically unknown. Moreover, the question on whether ORx, in addition to recognizing the odorant molecules, is also contributing to the formation of the pore is still open (Nakagawa et al., 2012).

Following the demonstration that ORco may form by itself *in vitro* a functional ion channel gated by the synthetic agonist VUAA1 (Jones et al., 2011), a number of related ORco agonists (OAs) were generated and their effects on homomeric ORco and heteromeric ORx/ORco channels assessed in various insects (Jones et al., 2011; Bohbot and Dickens, 2012; Chen and Luetje, 2012; Romaine et al., 2014). OAs were found to activate the ORx/ORco channels in the absence of odorant molecules (Jones et al., 2011; Chen and Luetje, 2012) and were also reported to synergize with odorant molecules and cause increased responses (Jones et al., 2011; Bohbot and Dickens, 2012; Rinker et al., 2012). However, the phenomenon of synergism between odorants and OAs has not been sufficiently explored. Nevertheless, these initial studies suggest that ORco may be a regulatory element of heteromeric ORx/ORco channel function.

While the effects of OAs on insect physiology, behavior and ecology have yet to be defined, their importance as tools for elucidating pharmacological features of the ORx/ORco channels is undisputed. Allostery is increasingly emerging as a most important regulatory feature of protein function, particularly from the viewpoint of impact on structure-function relationships in complex oligomeric assemblies (Hogg et al., 2005; Christopoulos et al., 2014; Langmead and Christopoulos, 2014), especially in cases, such as those of insect odorant receptors, where molecular structures are not available. From the different forms of allosteric regulation (Laskowski et al., 2009), the most common ones, pertinent to the present work, are those caused by (i) binding of small molecule effectors, and (ii) protein-protein interactions.

This study addresses issues related to allosteric regulation of selected members of *A. gambiae* ORs mediated by specific OAs belonging to the VUAA1/OrcoRAM2 family (Jones et al., 2011; Bohbot and Dickens, 2012). It focuses on aspects of allosteric modulation of ORco-dependent activity in the context of different ORx/ORco heteromeric complexes and on differences in ORx/ORco heteromer activation by cognate odorant ligands in the presence of the same OAs. The findings of this study are integrated into a model of proposed allosteric modulation of odorant-gated olfactory channel function. The results may also have implications for the development of new reagents for enhancement of insect responses to various odorants.

## Materials and Methods

### Chemicals

Odorants and OAs used in the current study are summarized in Supplementary Table S1. Linalool, hexanoic and octanoic acid, nonanal, and isoamyl alcohol were kind gifts from our colleagues in NCSR Demokritos, Drs Maria Konstantopoulou, D. Tsiourvas and G. Voutsinas. The OA VUAA1, used in preliminary experiments, was a generous gift of Professor R. D. Newcomb, New Zealand Institute for Plant & Food Research. Coelenterazine was obtained from different vendors: Promega, BIOMOL GmbH (Hamburg, Germany), Biosynth (Staad, Switzerland), and Carl Roth GmbH (Karlsruhe, Germany), while Triton X-100 was from Panreac. Initial stock solutions and dilutions for ORcoRAM2 were made in DMSO, while all remaining chemicals were diluted in methanol or ethanol. Working dilutions were freshly prepared immediately before use in modified Ringer buffer (190 mM KCl, 25 mM NaCl, 3 mM CaCl_2_, 3 mM MgCl_2_, 20 mM Hepes, 22.5 mM glucose, pH 6.5), which reflects more closely the composition of sensillum lymph (Kaissling and Thorson, 1980; Grünert and Gnatzy, 1987; Olsson and Hansson, 2013). Relative to the previously used Ringer buffer at pH 7.2 (Tsitoura et al., 2015), this buffer yielded much higher responses, with considerably higher signal-to noise ratios and retention of specificity, without adverse effects on cell viability (Supplementary Figure S1).

### Plasmids

The cDNAs encoding *A. gambiae* odorant receptors (Iatrou and Biessmann, 2008) and the calcium photoprotein Photina (Axxam SpA, Milan, Italy) were expressed in lepidopteran insect cells by the plasmid vector pIE1/153A [for brevity pEIA; (Lu et al., 1997; Farrell et al., 1998; Douris et al., 2006)]. The construction and use of pEIA.OR1, pEIA.OR2, pEIA.OR9, pEIA.ORco, and pEIA.Photina have been previously reported (Tsitoura et al., 2010; Tsitoura et al., 2015). The PCR amplification of the complete OR53 open reading frame (ORF) from antennal cDNA preparations, obtained as previously described (Iatrou and Biessmann, 2008), was carried out using forward and reverse primers OR53-FA/C [GAAT*GGATCC*CACC**ATG**AAGTTACTAGAGCTAGACAAC] and OR53-RA/N [GATA*GGATCC*TTAGAATACATTTTTTAGCACCAAG], respectively, (*Bam*HI restriction sites are in italics, initiation codon is in bold and termination codon is underlined). This was followed by subcloning in the *Bam*HI site of the pEIA vector as previously described (Douris et al., 2006; Tsitoura et al., 2010). Modified versions of pEIA (Douris et al., 2006) were used for N-terminal tagging of ORco with the FLAG epitope [(M)DYKDDDDK] (Tsitoura et al., 2010) and ORs 1, 2 (Tsitoura et al., 2010), OR9 and OR53 with the Myc epitope [(M)EQKLISEEDL]. For the Myc-tagged versions of OR9 and OR53 forward amplification primers GAAT*GGATCC*GTTAGGCTTTTCTTCAGCAAAAC and GAAT*GGATCC*AAGTTACTAGAGCTAGACAACC, respectively were employed with the reverse amplification primers used for the cloning of the authentic forms. All cloned sequences were determined upon initial cloning and also following their subcloning into the expression vector to ensure lack of nucleotide substitutions or other mutations arising from secondary PCR amplifications.

### Cell Culture and Transfection

*Trichoplusia ni* BTI-Tn 5B1-4 HighFive^TM^ cells (henceforth Hi5; Fisher Scientific) were used in the current study. The cells were grown in IPL-41 insect cell culture medium (Genaxxon Bioscience GmbH) with 10% fetal bovine serum (Sigma or Biosera) and maintained at 28°C. Transfection was performed with Escort IV (Sigma) or Biotool DNA transfection (Biotool) reagents, using 2 μg of total plasmid DNA and 6 μg of reagent per 10^6^ cells, according to the manufacturer’s instructions.

### Expression of Mosquito ORs and Bioluminescence Assays

The expression of *A. gambiae* ORs in lepidopteran insect cells and their functional characterization by luminescence assays have been previously reported (Tsitoura et al., 2010, 2015). Briefly, Hi5 cells were transfected with pEIA.ORx, ORco and Photina at per weight ratios (essentially molar ratios as well) of 1:1:2, or with pEIA.ORco and Photina or pEIA.ORx and Photina at 1:1. Cells were collected 2–4 days post-transfection, washed and resuspended in Ringer solution, after which coelenterazine was added at 5 μM and cells transferred to 96-well plates (200,000–300,000 cells/well) and incubated at RT in the dark for a minimum of 2 h. The Infinite M200 microplate reader (Tecan Group Ltd) was used for measuring luminescence, before and after application of odorants and OAs. Each experiment was performed in triplicates, and was repeated in independent experiments, as indicated for each figure. Results are presented as means ± standard deviation.

### ORco Agonism and Allosteric Modulation

To study direct agonism, the OA ORcoRAM2 or, in some cases, VUAA1, as exemplified in Supplementary Figure S2, was applied to cells expressing ORco alone, or the different ORx/ORco heteromers, or ORx alone (as negative controls), either at 100 μM or at increasing concentrations for the dose-dependent experiments. In some experiments that required comparisons of absolute values (i.e., magnitude of responses) between different heteromers and the ORco homomer, normalization for differences in transfection efficiencies and cell numbers was performed by permeabilizing cells with Triton X-100 detergent (up to 0.15%), or by measuring intracellular Ca^2+^-release responses in cells co-expressing a delta opioid receptor together with Gα16, following administration of its ligand DPDPE as previously described (Tsitoura et al., 2015). In such case, cells expressing ORco homomers or ORco/ORx heteromers were challenged sequentially with OA and TX100 in a separate series of wells, in order to avoid artifacts from desensitization and responses were calculated as ratios of OR response/TX100-evoked increase in luminescence. The same was the case for co-expression with the delta opioid receptor, except that the delta opioid receptor agonist DPDPE was used instead of TX100 and responses were normalized as OR response/opioid receptor response. To study the synergistic effect of OAs and odorants, 5–10 μM concentrations of ORcoRAM2 or VUAA1 were applied before, after or simultaneously with a chosen concentration of odorant of interest, and responses were measured in the microplate reader.

### Data Analysis and Curve Fitting

Data acquisition was performed with i-Control 1.3 (Tecan). As before (Tsitoura et al., 2015), luminescence value comparisons between independent experiments were made relative to normalization standards. Thus, the specific agonist (odorant or OA) at highest concentration or both (for potentiation experiments) were considered to cause 100% (maximal) response for the specific set of experiment. For curve fitting and EC_50_ calculations (expressed also in the form of the negative logarithm of the EC_50_, pEC_50_, which gives a commensurate measure of potency), GraphPad Prism 4.0^1^ was used. Specifically, concentration-response data were fitted to the equation for non-linear regression, sigmoidal dose-response (variable slope): *Y* = Bottom + (Top – Bottom)/(1 + 10ˆ(LogEC_50_ – *X*)*Hillslope), where *Y*: % response at a given concentration; *X*: logarithm of concentration, with Top and Bottom values being the maximal and minimal % responses and the following constraints being applied: bottom >0.0 and Top <100.0. Each independent experiment was performed in triplicate wells, with the number of repetitions indicated for each graph. Statistical analysis was one-way ANOVA followed by Bonferroni’s multiple comparison test.

### Antibodies and Western Blot Analysis

Expression of ORs by western blot analysis was essentially as previously described (Tsitoura et al., 2010). In particular, Myc-tagged ORs 1, 2, 9, and 53 were detected in total lysates of transiently transfected Hi5 cells, by the use of mouse anti-Myc antibody (Cell Signaling 9B11, 1:1,000 dilution). For Flag-tagged ORco protein detection in cells expressing either ORco (fORco) alone or its combinations with various ORx subunits, the anti-Flag antibody (Sigma F1804, 1:800 dilution) was used. In the latter case, Hi5 cells were transfected with constructs directing expression of fORco, OR1/fORco, OR2/fORco, OR9/fORco or OR53/fORco and Photina, and cells were used for western blot analysis and, in the presence of co-expressed Photina, functional assays.

### Sequences and Predictions

The sequencing of the clones for the OR9 and OR53 receptor subunits, which were employed in this study, revealed differences from their previously reported counterparts (VectorBase IDs AGAP008333-PA and AGAP009390-PA, respectively). These consisted of segmental insertions of 9 and 8 amino acids, respectively (NCBI Accession numbers KX697339 and KX697340, respectively). These were apparently produced as a result of differential splicing of the respective primary transcripts, which gave rise to the specific mRNAs shown in Supplementary Figures S3A,C, respectively. The segmental insertions were predicted to reside in the corresponding second intracellular and second extracellular loops (Supplementary Figures S3B,D, respectively). Transmembrane (TM) domain predictions for OR9 and OR53 were made using the TMpred program^2^ and TOPCONS^3^ (Tsirigos et al., 2015), and the schematic drawings in Supplementary Figures S3B,D (right) were generated based on results from the latter. Predicted TM topologies for OR53 (TOPCONS) were 33–53, 68–83, 123–143, 174–194, 252–272, 281–301, 354–374. For OR9, six TM regions were predicted with most TOPCONS sub-methods used. The OCTOPUS and SPOCTOPUS sub-methods, however, predicted seven TM domains at 56–76, 87–107, 148–168, 192–222, 288–308, 319–339, 389–409 with the last one being predicted only by these two algorithms.

## Results

### ORco Agonist-Induced Activation of Olfactory Receptor Heteromers

The effects of OAs on the function of *A. gambiae* olfactory receptor heteromers were examined in lepidopteran insect cells expressing either ORco or different ORx/ORco heteromers and the reporter photoprotein Photina, as previously described (Tsitoura et al., 2015). Four ORx subunits were tested in this study, OR1, OR2, OR9, and OR53, all displaying high female antenna-biased expression (Iatrou and Biessmann, 2008). OR1 and OR2 have been selected as specialist receptors responding to chemicals of great importance for mosquito physiology, including oviposition cues and components of human sweat (Hallem et al., 2004; Carey et al., 2010; Wang et al., 2010). OR9 and OR53, on the other hand, were used as examples of other deorphanized receptors that displayed notably lower responses in various functional assays (Carey et al., 2010; Wang et al., 2010). Indicatively, in the *Xenopus* system, response magnitudes of 100–150 were reported for OR9 and OR53 against their cognate ligands, while by comparison, response magnitudes of OR2 and OR4 against their ligands were up to 600 and those for OR10 and OR28 were in the range of 3000–4000 (Wang et al., 2010). Although functionally analyzed to a limited extent, these latter receptors have not been subjected to detailed pharmacological characterization.

The functionality of ORco homomers and OR1/ORco and OR2/ORco heteromers with their ligands in the specific insect cell-based assay has been demonstrated previously (Tsitoura et al., 2015). The OR9 and OR53 isoforms employed in this study (Supplementary Figure S4) were also found to be functional in the same assay. Thus, the OR9/ORco heteromeric receptor responded to 2-ethylphenol and to a much lesser extent to 4-methylphenol and 3-methylphenol (Supplementary Figure S4A, left), in agreement with the previously established odorant selectivity (Carey et al., 2010; Wang et al., 2010). For 2-ethylphenol, a dose response analysis revealed an EC_50_ of approximately 78 μM (Supplementary Figure S4A, right; **Table 1**). On the other hand, the OR53/ORco receptor was functionally tested with a number of odorants that were selected based on previous studies, which reported functional responses in either the *Xenopus* oocyte or the *Drosophila* empty neuron models (Carey et al., 2010; Wang et al., 2010), while keeping in mind that some of these chemicals yielded contradictory results in the two systems. From the tested chemicals, we obtained clear responses only with linalool (Supplementary Figure S4B), a compound related to linalool oxide, which was reported to be slightly active against this receptor in the empty neuron system (Carey et al., 2010). The responses to linalool were found to be dose-dependent (data not shown), however, its EC_50_ at ∼180 μM was comparable to those of OR1 and OR2 partial agonists in this system, 3-methylphenol (for OR1/ORco) and benzaldehyde and 2-methylphenol (for OR2/ORco) (Tsitoura et al., 2015 and data not shown). Nevertheless, additional functional assays described below established firmly the functionality of this receptor.

**Table 1:** EC_50_ values from concentration-dependent response curves.

Receptor	Chemical	EC_50_ (pEC_50_ ± SE) R^2^	Reference
OR1+ORco	OA	56.6 μ M (4.247 @ 0.05688) 0.9831	**Figure 2**
	4MP	2.8 μ M (5.548 @ 0.1984) 0.9416	Tsitoura et al., 2015
		1.2 μ M (5.927 @ 0.03852) 0.9961	**Figure 4**
	4MP+OA	104.6 n M (6.981 @ 0.2329) 0.9772	**Figure 4**

OR2+ORco	OA	43.6 μ M (4.361 @ 0.02894) 0.9903	**Figure 2**
	IN	3.4 μ M (5.465 @ 0.1186) 0.9829	Tsitoura et al., 2015
		5.5 μ M (5.258 @ 0.03010) 0.9865	**Figure 4**
	IN+OA	52.5 n M (7.280 @ 0.1809) 0.9667	**Figure 4**

OR9+ORco	OA	78.7 μ M (4.104 @ 0.01060) 0.9973	**Figure 2**
	2EP	77.6 μ M (4.110 @ 0.009062) 0.9992	**Figure 4**; Supplementary Figure S4
	2EP+OA	6.1 μ M (5.213 @ 0.08414) 0.9845	**Figure 4**

OR53+ORco	OA	56.6 μ M (4.247 @ 0.03210) 0.9906	**Figure 2**
	LIN (partial agonist)	181.1 μ M (3.742 @ 0.04453) 0.9944	**Figure 4**
	LIN+OA	12.6 μ M (4.899 @ 0.1493) 0.9800	**Figure 4**

ORco	OA	58.9 μ M (4.23 @ 0.034) 0.9655	Tsitoura et al., 2015
		96.6 μ M (4.015 @ 0.02398) 0.9863	**Figure 2**

*Summarized are the EC_50_ values obtained from curves presented in **Figures 2** and **4** and Supplementary Figure S4. For the four ORx/ORco heteromeric complexes studied (with OR1, OR2, OR9, and OR53), the numbers presented here concern EC_50_ values of specific odorants (4MP, IN, 2EP, and LIN, respectively), ORco agonist ORcoRAM2 (OA) and the specific combinations used in potentiation studies. EC_50_ values of OA for the homomeric ORco channels are also presented. EC_50_, half maximal effective concentration; pEC_50_, negative logarithm of the EC_50_ (–logEC_50_); Std. error, standard error (SE) reported by GraphPad Prism for calculated logEC_50_; R^2^, measure of goodness of fit. Previously obtained values for OR1/ORco (4MP), OR2/ORco (IN), and ORco (OA) (Tsitoura et al., 2015) are also listed.*

**FIGURE 1:**
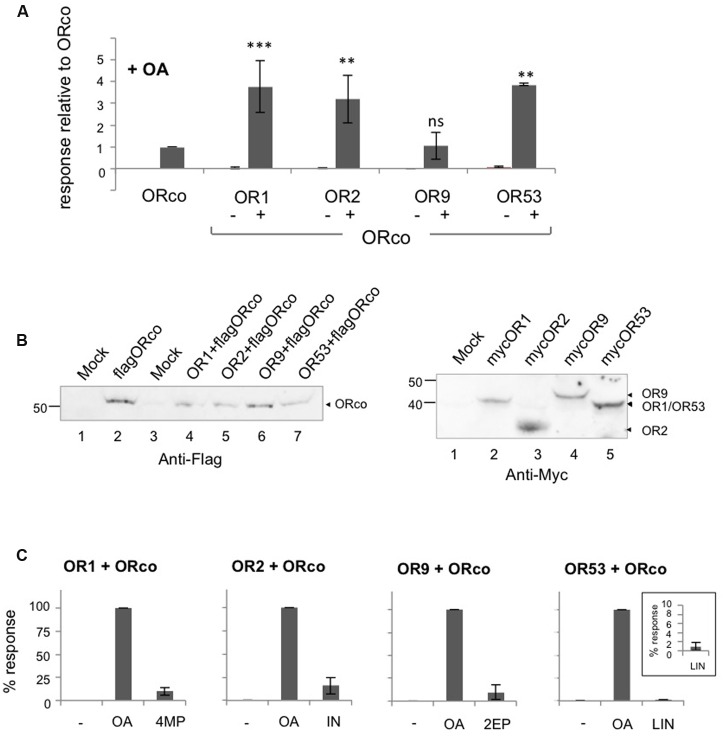
**Direct agonism of heteromeric and homomeric OR receptor function by ORco agonists (OAs).** Results of experiments employing OrcoRAM2 as an OA are shown. **(A)** Magnitude of responses obtained from cells expressing ORco, OR1, OR2, OR9, OR53 alone or the same ORx subunits as heteromers with ORco, in the presence of 100 μM of the OA. (*n* = 2 for OR9, OR53, and OR53/ORco; 3 for OR1, OR2, OR9/ORco; 6 for OR1/ORco, OR2/ORco, and ORco alone, the latter being tested in all experiments). Positive responses only were assessed by one-way ANOVA, followed by Bonferroni’s multiple comparison test, and significances of each heteromer’s response relative to ORco are depicted: ^∗∗^*P* < 0.01; ^∗∗∗^*P* < 0.001. **(B)** Left. Detection of ORco by western blot analysis in cells expressing ORco homomeric or ORx/ORco heteromeric complexes. Flag-tagged version of ORco was used in this experiment, and detection was performed by monoclonal antibody against the Flag epitope. Right. Detection of expression of ORs 1, 2, 9, and 53. Myc-tagged versions of ORx subunits were used in this experiment, and detection was performed by monoclonal antibody against the Myc epitope. The mock sample contains lysates from untransfected cells. **(C)** Comparison of the magnitudes of the heteromers’ responses to their specific ligands (SLs) (4MP for OR1, IN for OR2, 2EP for OR9, and LIN for OR53) relative to those obtained with the OA, both applied at a concentration of 100 μM. The inset in the bargraph for OR53 presents more clearly the low response of the cells that express the OR53/ORco heteromer to the partial agonist LIN (*n* = 3, 6, 4, and 2 for ORs 1, 2, 9, and 53, respectively).

To investigate the effects of OAs on the different odorant receptor heteromers, we examined the functional responses of lepidopteran cells expressing the respective receptor subunits upon addition of VUAA1 or OrcoRAM2, both of them members of the first reported class of OAs. As shown in **Figure 1A**, unequivocal responses, in terms of Ca^2+^-ion entry, could be detected in cells expressing ORco homomers and its heteromers with OR1, OR2, OR9, and OR53 upon treatment with 100 μM of OrcoRAM2, a concentration equal to the EC_50_ for ORco (**Table 1**).

For three of the four ORx/ORco receptor heteromers examined, the responses triggered by the OA were more robust, on average 3 to 4-fold (**Figure 1A**; Supplementary Table S2), than those observed in cells expressing ORco alone (**Figure 1A**), even though the latter contained nearly double the amount of ORco relative to cells expressing each heteromer due to respective differences in ORco expression vector quantities transfected into the cells (**Figure 1B**, left). OR9 was the only ORx not to conform to this observation but this may have been due to a consistently lower quantity of receptor expressed in the transfected cells, at least relative to OR2 and OR53; additional factors, however, may account for this, as levels of OR9 expression are quite similar with those of OR1 (**Figure 1B**, right). Importantly, the efficacies of the responses of the tested receptors to the specific dose of OA were also strikingly higher than those observed with an equivalent concentration (100 μM) of ORx-specific agonists (**Figure 1C**). This concentration of ORx-specific ligands (SLs) represented a range of EC values [nearly EC_90_ for 4-methylphenol and indole against OR1 and OR2, respectively (Tsitoura et al., 2015); EC_60_ for 2-ethylphenol against OR9 (Supplementary Figure S4A; **Table 1**); and ∼EC_18_ for linalool against OR53 (**Table 1**)]. The latter increases in receptor heteromer response efficacies ranged from 6-fold to 10-fold for three of the four tested receptor heteromers (those of OR1, OR2, and OR9), while for the OR53 heteromer the response to the OA was more than 100 times more efficacious than linalool (**Figure 1C**; Supplementary Table S2). Considering, however, that 100 μM of linalool, which is a partial OR53 agonist, represented only an ∼EC_18_ value for OR53, the latter difference was not unexpected. However, the observed enhanced efficacy of the OA versus the odorant is maintained or even increased, when concentrations of OA and odorants are adjusted for each receptor heteromer, to be more close to equipotency (as shown for representative OR members in Supplementary Figure S5).

**FIGURE 2:**
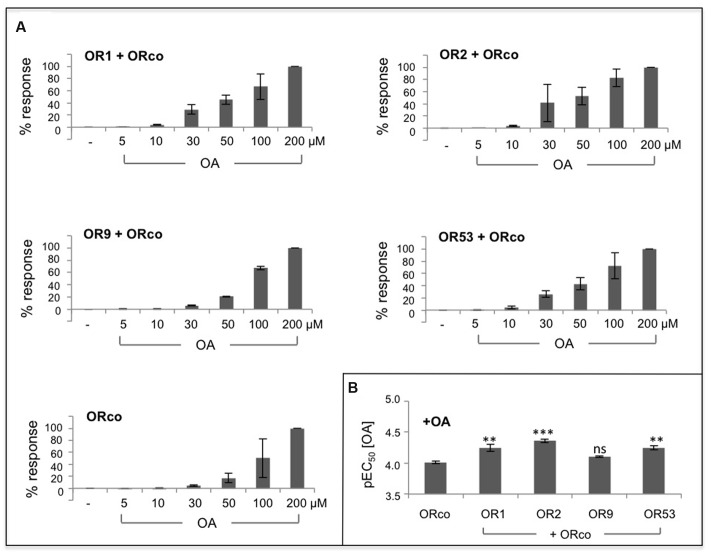
**Dose-dependent agonism of heteromeric receptor function by OAs. (A)** Functional responses of cells expressing ORco alone or OR1/ORco, OR2/ORco, OR9/ORco, and OR53/ORco heteromers to increasing concentrations of OrcoRAM2. Results shown are from 3 to 4 independent experiments, each performed in triplicates; the responses for each receptor are normalized to the highest one (100%) obtained with 200 μM of the OA. **(B)** Comparison of the pEC_50_ values for ORco homomer and the four studied heteromers (^∗∗^*P* < 0.01; ^∗∗∗^*P* < 0.001, for each heteromer relative to ORco).

An OA dose response analysis for the studied receptor heteromers showed that, similar to the case of the ORco homomer, administration of low concentrations of OrcoRAM2, in the order of 5–10 μM, to cells expressing the specific heteromers produced only minimal, if any, responses (**Figure 2A**). However, in the presence of slightly higher concentrations of OA, in the order of 30 μM, ORx/ORco heteromers yielded noticeably higher functional responses than the ORco homomers (**Figure 2A**). Nevertheless, as shown in **Figure 2B**; **Table 1**, the OA dose responses for OR1, OR2, OR9, and OR53 heteromers revealed very similar OA potencies (EC_50_ values of 57, 44, 79, and 57 μM, respectively) relative to the ORco homomers (EC_50_ of 59 and 97 μM in two independent studies; **Table 1**).

These results suggest that the enhanced functional responses of the tested ORx/ORco heteromeric channels relative to ORco homomers upon OA administration are probably due to conformational changes and consequential activity increases induced on the ORco channel as a result of its association with the specific ORx subunits. An alternative explanation invoking increased stability of ORco or even enhanced partitioning on the cell plasma membrane due to its association with ORx subunits in the context of the heteromer as cause for the enhanced functional responses of the heteromeric channels to the OA, cannot be excluded without further experimentation.

Whether the interaction of ORco with ORx subunits results in the generation of new channel pores formed with contributions by both ORco and ORx, is not possible to deduce from these results alone. Nevertheless, given the similarity of OA potencies for ORco homomeric and heteromeric channels, the simplest scenario would predict the existence of a common, ORco-based channel pore whose structure and activity are regulated by changes induced by the interactions with the different ORx subunits. The fact that, for any given heteromer, the response to the OA appears to be more efficacious than the response to each specific odorant is also a significant finding as it suggests that each heteromer can exist in different conformations leading to differential functional outputs.

### ORco Agonist Acts as Enhancer of ORx Ligand-Induced Responses

To assess whether the presence of an OA may also affect the ORx ligand-dependent responses, cells co-expressing different heteromeric receptors were treated with 10 μM OrcoRAM2, a concentration that is by itself essentially unable to trigger substantial responses (**Figure 2A**), prior to or concurrently with the addition of 100 μM of various ORx cognate odorants.

**FIGURE 3:**
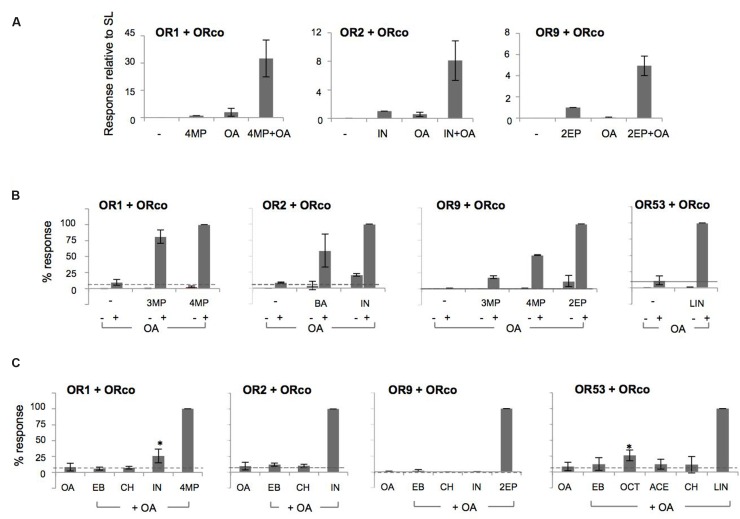
**OAs act as allosteric enhancers of heteromeric receptor function. (A)** Cells expressing OR1/ORco, OR2/ORco, and OR9/ORco were challenged with 100 μM of their specific agonists (4MP, IN, and 2EP, respectively) in the absence (–) or presence (+) of a low concentration (10 μM) of OA (ORcoRAM2). OA-dependent potentiation was observed for all three tested heteromers (*n* = 5, 6, and 3, respectively). **(B)** Potentiation of responses to 100 μM of partial agonists (3MP for OR1/ORco, BA for OR2/ORco, 3MP and 4MP for OR9/ORco, and LIN for OR53/ORco) by 10 μM of the OA. Individual application of odorants alone is depicted by – signs, while the combined application of the OA and odorants is indicated by + signs (*n* = 2 for ORs 1, 2, and 9; *n* = 5 for OR53). **(C)** Lack of measurable potentiation by cells expressing OR1, OR2, OR9, and OR53 heteromers with ORco upon administration of odorants that do not normally activate these receptors and a low concentration (10 μM) of the OA. In all experiments, the SLs (4MP for OR1, IN for OR2, 2EP for OR9, and the partial agonist LIN for OR53) were used as positive controls providing maximal (100%) OA-potentiated responses (*n* = 3 for OR1 and OR2; 2 for OR9; and 3–5 for OR53). The cases of IN (for OR1) and OCT (for OR53), which appear as apparent exceptions to the behavior of odorants not recognized by the respective receptor heteromers are discussed in the main text (significance of OA+IN relative to OA and OA+OCT relative to OA for OR1 and OR53, respectively, is depicted: ^∗^*P* < 0.05).

As may be seen in **Figure 3A**, a very strong enhancement in specific odorant-induced responses was obtained from cells treated with the low concentration of OA relative to the responses obtained in its absence. The relative increases in response magnitudes ranged from 5 to more than 30-fold for the receptors with known agonists (OR1, OR2, and OR9; Supplementary Table S2). The potentiation of the receptors’ responses by 10 μM OA, at equipotent, EC_20_, concentrations of odorants for the different heteromers, was also strong, ranging from 10 to almost 95-fold (Supplementary Figure S6). On the other hand, the responses of the cells to the partial ORx agonists, 3-methylphenol (for OR1), benzaldehyde (for OR2), 3- and 4-methylphenol (for OR9) and linalool and octanoic acid (for OR53) [(Wang et al., 2010; Tsitoura et al., 2015); and Supplementary Figure S4A] were also enhanced considerably by the addition of the low concentration of OA. However, relative to the respective best agonists, the OA-induced potentiation of partial agonist responses was distinctly lower (**Figure 3B**). A possible qualification for this conclusion concerns OR53, for which a 140-fold enhancement was observed (up to 250 for the EC_20_ concentration of linalool, Supplementary Figure S6), because the best cognate ligand for this receptor has yet to be determined and, therefore, a direct comparison could not be made. Importantly, no activity induction by the low concentration of the OA was obtained with compounds that do not normally activate ORx/ORco receptors (**Figure 3C**). We also note that the OA-induced potentiation of responses occurred irrespective of the order of addition of the OA to the cells relative to the administration of the ORx agonists (data not shown).

These results suggest that although the OA augments the responses of the ligand-gated OR channels to their physiological agonists, it does not alter the existing affinity differences between agonists. Two notable exceptions to this rule were the cases of indole for OR1 and octanoic acid for OR53, which yielded responses in the presence of the low OA concentration despite the fact that they did not act as specific agonists, even partial ones, in our system. For OR1, the lack of indole recognition has been documented in all testing systems used for functional characterization (Xia et al., 2008; Carey et al., 2010; Wang et al., 2010; Tsitoura et al., 2015). For octanoic acid, activity against OR53 has been shown in *Xenopus* oocytes (Wang et al., 2010) but not the *Drosophila* empty neuron system, where it was found to be inactive (Carey et al., 2010), or our system (up to a concentration of 100 μM; Supplementary Figure S4B). Whether these chemicals may activate partially the respective receptors at higher concentrations in insect cell-based system has not been examined.

**FIGURE 4:**
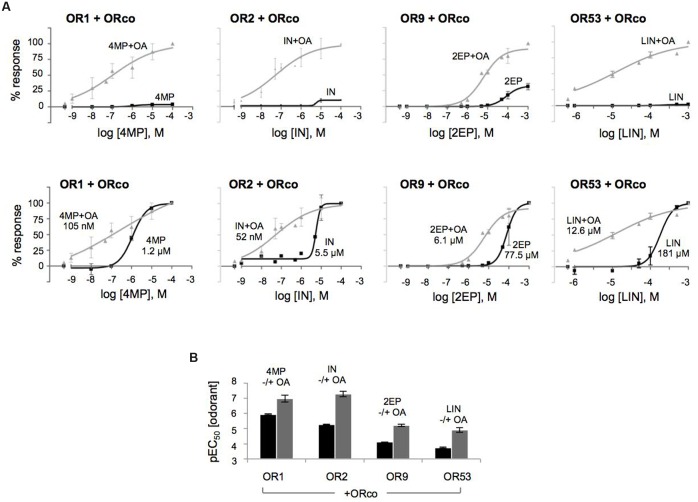
**The OA is an allosteric modulator that enhances both the affinity and efficacy of odorant recognition by cognate olfactory receptors *in vitro*. (A)** Upward and leftward shifts of the dose-response curves of OR1, OR2, OR9, and OR53 heteromers with ORco, to their SLs 4MP, IN, 2EP, and (partial agonist) LIN, respectively, in the presence of a low concentration of OA (10 μM). The upper and lower panels present the same results with different types of normalization: in the upper panels, the responses for each receptor were normalized relative to the highest responses (100%) obtained with each SL in the presence of 10 μM of the OA (SL+OA); while in the lower panels, the responses for each receptor were separately normalized to the maximum value (100%) obtained at the highest concentration of each SL alone or in the simultaneous presence of 10 μM of the OA (SL+OA) to make more evident the leftward shift. **(B)** Comparison of the pEC_50_ values of the specific odorants alone or together with 10 μM of the OA (black and gray bars, respectively) for the four tested heteromers. The pEC_50_ values (also listed in **Table 1**) are (mean ± SE): 4MP 5.927 ± 0.03852 and 4MP+OA 6.981 ± 0.2329 for OR1/ORco; IN 5.258 ± 0.03010 and IN+OA 7.280 ± 0.1809 for OR2/ORco; 2EP 4.110 ± 0.009062 and 2EP+OA 5.213 ± 0.08414 for OR9/ORco; and LIN 3.742 ± 0.04453 and LIN+OA 4.899 ± 0.1493 for OR53/ORco. (*n* = 2 for OR1/ORco and OR9/ORco, 3 for OR2/ORco and OR53/ORco).

To obtain a more detailed assessment of the potentiation of olfactory receptor responses by the low concentration of the OA, dose response curves were constructed for the specific ligands (SLs). As shown in **Figure 4**, the presence of OrcoRAM2 caused a notable, ORx ligand-dependent enhancement in the functionality of the heteromeric receptors of known odorant ligand recognition both in terms of efficacy and potency. Specifically, while the increases in response magnitudes of the tested receptors ranged from 6-fold to 32-fold (and 141-fold relative to linalool for OR53) (**Figure 4A**; Supplementary Table S2), the potency increases (decreases in EC_50_ values) ranged from 12-fold to more than 100-fold (**Figure 4B**; **Table 1**). The shifts of the curves, as well as the concomitant potency changes in EC_50_ values were intermediate when a lower concentration (1 μM) of OA was used instead of 10 μM (data not shown).

Collectively, these results provide support to the notion that OAs, even when present at low concentrations, induce allosteric changes to odorant-gated heteromeric receptor channel structures resulting in significant positive modulation of responses to specific odorants. These allosteric changes appear to be also ORx ligand-dependent.

## Discussion

ORco, the insect olfactory co-receptor, is steadily becoming the focus of attention because of discoveries linking its functionality to new insights into the function of olfactory receptors and new directions in olfaction-based approaches for insect pest control. Its importance has been already documented, mainly through studies employing specific RNAi to suppress ORco expression (Yi et al., 2014; Zhou et al., 2014; Fan et al., 2015; Lin et al., 2015; Franco et al., 2016; Zhang et al., 2016) or complete elimination of Orco *via* genome editing (DeGennaro et al., 2013; Koutroumpa et al., 2016). Additionally, we have previously established that a series of strong mosquito repellents act as ORco antagonists that cause the *in vitro* blocking of specific odor recognition by olfactory receptors requiring its presence for functionality (Tsitoura et al., 2015). Moreover, several other compounds were also shown to be capable of inhibiting insect odorant receptor function through antagonism of the co-receptor subunit (Chen and Luetje, 2012; Jones et al., 2012; Chen and Luetje, 2013; Pask et al., 2013; Chen and Luetje, 2014). In the current report, we present results suggesting that OAs may be also used as agents that enhance the odor recognition sensitivity of the mosquito olfactory system.

The finding that ORco may act as a ligand-gated channel in the absence of ORx subunits (Jones et al., 2011) has been a major discovery for the field. However, the question of whether ORco homomers may actually exist in olfactory receptor neurons remains unanswered. Should ORco homomers and ORx/ORco heteromers co-exist in olfactory receptor neurons and assuming that natural OAs also exist that act on ORco in the same fashion as VUAA1 and OrcoRAM2, based on the *in vitro* results presented here (**Figure 1A**) we would predict that the heteromeric channel responses to them would be considerably stronger than those of any co-existing ORco homomeric channels. Therefore, the overall response profiles of the heteromeric population should not be influenced significantly by any co-existing ORco homomers.

Because of their large size and essential lack of volatility, the available synthetic OAs (Chen and Luetje, 2012; Romaine et al., 2014), including those used in the current study, may not be of physiological relevance. Nevertheless, they constitute useful tools for the pharmacological characterization of olfactory receptors. For example, they may be used, as was shown in this study, for activity comparisons between ORco homomers and ORx/ORco heteromers, as well as direct pharmacological comparisons of different ORx/ORco heteromers without interference from effects exerted by specific ORx ligands. In this context, the first major finding of this study has been that in the absence of ORx cognate ligands, relatively high concentrations (100 μM) of the specific OA ORcoRAM2 trigger, in most examined cases, heteromeric channel responses whose magnitudes are noticeably higher than those of the ORco channels (**Figure 1**; Supplementary Table S2) essentially without major concomitant changes in agonist potencies (**Figure 2**; **Table 1**). Both of these findings may be explained by the induction of differential conformational changes on ORco homomeric channels caused by the interacting ORx subunits. Alternative explanations, however, cannot be excluded in the absence of further experimentation. For example, it is conceptually possible that the association of ORco with ORx subunits results in an increased stability of heteromeric complexes relative to homomeric ones or even increased partitioning on the cellular plasma membrane.

**FIGURE 5:**
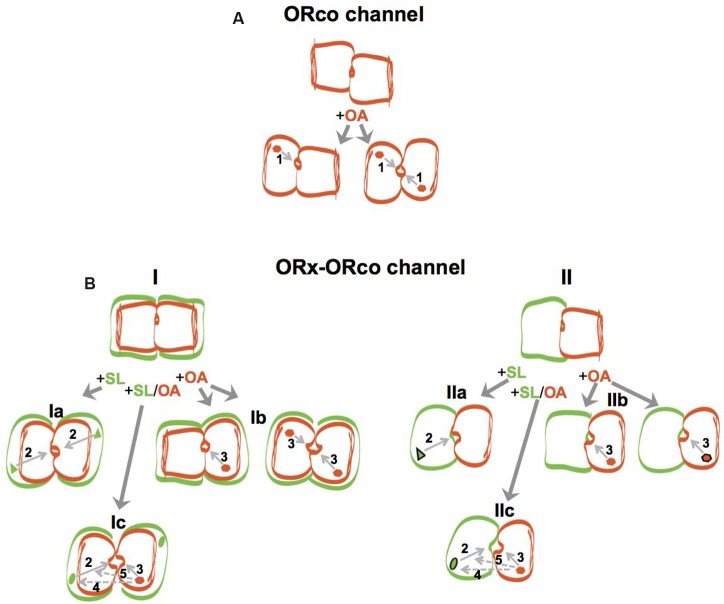
**Models of ORco homomeric and ORx/ORco heteromeric channels and schematic overview of allosteric modulation by OAs.** Channel subunits are indicated by rectangular or oval shapes of different colors (brown for ORco and green for ORx), with conformational changes induced on subunits as a result of ORco or ORx-specific agonist binding being indicated by changes in shape (from rectangular to oval) and channel pore openings of various sizes induced by different ligands indicating magnitudes of ion permeability. ORco and ORx ligands are indicated by brown hexagons and green triangles or green ellipsoids, respectively, inside the corresponding receptor subunits. For all cases, conformational changes occurring in any given channel subunit have notable effects on the structure of its interacting subunit due to altered protein-protein interactions, and induce changes in magnitudes of ion permeability indicated by commensurate changes in channel pore sizes. **(A)** **ORco channel:** the different states of the homomeric ORco channel, which is presented here as a dimer, are shown with the unliganded, inactive state indicated by misaligned subunits and a closed pore, and the partially (+OA left) or fully active (+OA right) states indicated with the partly or fully changed shapes of one or both channel subunits, depending on agonist concentrations, alignment of channel subunits and commensurately increasing pore widths. Arrow 1 indicates the final effect of ligand gating on the channel pore. **(B)** **ORx/ORco channel:** the different states of the heteromeric channels (ORx/ORco channel) are shown, with dimers and tetramers illustrated for simplicity. Schematic in **(I)** shows heteromers containing an ORco-based channel pore, while **(II)** illustrates heteromers with the channel pore formed with contributions by both ORx and ORco subunits. For **(I)**, a low concentration of the OA is indicated by a single binding site per receptor complex (**Ib**, left), while ORco and ORx-specific agonist concentrations at or higher than EC_50_ are indicated by two bound agonists per receptor complex (**Ib**, right, and **Ia**). In the case of **(II)**, the high ORco or ORx agonist states are indicated with the respective ligands being highlighted with black contour (**IIb**, right, and **IIa**), while the enhanced potency of the ORx agonist resulting from the simultaneous binding of the OA, observed under incomplete agonist occupancy conditions, is indicated by the change of the ligand’s shape from triangle to ellipsoid and the presence of the black contour **(IIc)**. Arrows 2 and 3 indicate the effects of specific ORx ligands or the OAs, respectively, on channel pore permeabilities, while arrows 4 and 5 (with dotted lines) point to the elements on which the OA is hypothesized to act and thus affect the nature of the binding pocket (potency) and efficacy, respectively, of the SL. Note that for reasons of figure clarity, only one set of the predicted interactions is illustrated in the case of the high ligand concentration **(Ic)**.

A second important finding, shown for all combinations studied here, has been that the specific OAs synergize with ORx-SLs and act as positive allosteric modulators of heteromeric channel function. The potentiation of responses obtained *in vitro* from the olfactory channels upon administration of ORx-SLs in the presence of low concentrations of an OA had been noted previously (Jones et al., 2011; Bohbot and Dickens, 2012) but not studied in detail. The conformational changes that apparently are induced by the OA on the heteromeric agonist-gated complexes, even at the low concentration of 10 μM employed in our study, which by itself provides no or only minor functional competence to homomeric and heteromeric channels *in vitro* (**Figure 2**), account for the increases in both the response magnitudes evoked by the specific ORx agonists and their potencies (**Figure 4**). Interestingly, the potentiation exerted by the OAs on ligand-gated olfactory channel responses appeared to leave the fundamentals of ORx-ligand recognition specificity and function essentially unaltered, with highly potentiated responses obtained with the most potent agonist for any given receptor, somewhat lower responses achieved with less potent agonists, and no potentiation occurring with chemicals that do not activate the tested receptors (**Figure 3**). Based on the combined results of potentiation, we suggest that the use of low concentrations (10 μM or lower) of OAs in ligand screening programs may provide significant advantages both in terms of enhancement of responses that are at the threshold of detection and capacity to use lower concentrations of the screened compounds. In this regard, we note that in addition to the 10 μM OA concentration that we have presented and discussed here, we have also seen potentiation with 1 and 5 μM of ORcoRAM2 (data not shown). In view of the current paucity in our knowledge concerning the molecular structure of insect olfactory receptors, the results of this study also provide tantalizing suggestions concerning the modulation and certain mechanistic aspects of the activation of the heteromeric channels by odorants, OAs or both, and these are summarized in **Figure 5**. In this regard, a consideration of anticipated basic features of the homomeric and heteromeric OR channels may be instructive. Starting from **ORco** (**Figure 5A**), while the channel remains impermeable to ions in the absence of OA (non-aligned, brown rectangular subunits), a “channel on-channel off” (incomplete agonist occupation; brown hexagon) situation resulting from transient changes in the structure of the homomer (illustrated by the change from a rectangular to an oval-shaped subunit in **Figure 5A**), should exist at agonist concentrations 10-fold lower than EC_50_ (brown hexagon). These, apparently result in very low net influx of cations into the cells through the pore (arrow 1). In the presence of OA concentrations approaching or exceeding the EC_50_ for ORco (100 μM; full agonist occupation illustrated by brown hexagons in both subunits) [(Tsitoura et al., 2015); **Table 1**], the “channel on” (full agonist occupation) state should be favored, due to the stabilization of the structural changes in the homomeric channel (transition to structure with aligned oval subunits), which translate into increased rates of ion influx through the pore.

For the heteromeric channels (**Figure 5B**, **ORx/ORco channel**), two types of channel structure formation are envisaged irrespective of stoichiometric considerations (for simplicity, dimers and tetramers are illustrated for the heteromers in **Figure 5B** but other configurations are, of course, possible). Of the two possible alternatives, the first, which we consider more likely, predicts the existence of a channel structure analogous but not identical to that of the homomeric channel. This is formed again by the ORco subunits alone (**Figure 5B, I**), but its structure is affected (alignment of the brown rectangular subunits) because of the interactions of ORco with the ORx subunits (green rectangular subunits). The second type of channel envisages the generation of completely different type of channel pore formed with contributions from both ORx and ORco partner subunits (**Figure 5B, II**). In both cases, a positive regulatory role is predicted for the unliganded ORx subunits *via* their interactions with ORco and the induction of ORx-dependent changes to the ORco subunit structure (illustrated by the subunit alignment in **Figures 5B, I** and **II**). The latter, impact on the functional potential of the respective heteromeric channels and the permeability of their pores upon ligand addition, as well as their differential responses to OAs; however, potential effects of heterodimerization on stability and trafficking should also be born in mind.

For the responses of heteromeric channels to ORx cognate ligands, the binding of an ORx-SL to the ORx subunit (green triangles in **Figures 5B, Ia** and **IIa**) is envisaged to induce secondary, ORx subunit-mediated changes to the heteromeric structure (transitions to oval-shaped subunits in **Figures 5B, Ia** and **IIa**) contributing to the opening of the pore (arrow 2). For the functional responses of heteromeric channels to the OA in the absence of ORx cognate ligands (**Figures 5B, Ib** and **IIb**), we suggest that they follow the same fundamental principles noted for homomeric ORco channels but with the added feature of the conformational changes in channel structure induced by the interactions between ORx and ORco subunits, which impact positively on the permeability of the pores (arrow 3) thus causing an increase in OA efficacy relative to the homomeric channel. A caveat to this hypothesis is the previously mentioned possibility of stabilization or increased availability of ORco on the cell membrane due to its association with ORx subunits in the heteromeric complex.

For the potentiation of ORx ligand-gated heteromeric channel function by low concentrations of OAs (**Figures 5B, Ic** and **IIc**), we suggest that the allosteric changes effected on the heteromeric channel subunits by their interactions and those triggered by their respective agonists, synergize to cause a further increase in the “opening” of the channel and the rates of cation influx into the cells relative to the rates obtained by the specific ORx agonists or the high concentrations of OAs alone. Specifically, besides the allosteric effects discussed above (indicated by the transition to the oval subunit shape in **Figure 5B**), our results suggest that the binding of the OA to the ORco subunit may also enhance allosterically the binding of ORx-SL (arrow 4) by changing the properties of its binding pocket (green oval-shaped ligands) causing an increase in the potency and efficacy (arrow 5) of the ORx ligand, the latter in terms of magnitude of pore permeability induced by it. These postulates are compatible with the observation that the OA leaves the fundamentals of ORx ligand recognition specificity and function of the heteromeric channels essentially unaltered, with the potentiation of responses being proportional to the “native” (SL-induced) strength of the channel (**Figure 3**). For the synergistic action and activity changes induced upon simultaneous addition OA and odorant, we also note the possibility that enhanced stabilization (but not trafficking) of ORx-ORco heteromers may also be a contributing factor. This possibility should be formally addressed in future studies.

A distinction between the two models described above, both in terms of channel subunit stoichiometries and the nature of the channel pore, should be feasible despite the current lack of determined structures and the paucity of available information in existing computational structure predictions (Carraher et al., 2015; Hopf et al., 2015). For the stoichiometries of ORco homomer and ORx/ORco heteromer subunits, cross-linking studies should provide relevant clues, which could be further tested by mutational studies to identify residues critical for subunit interface interactions. For the issue of the nature of the heteromeric channel, on the other hand, differential labeling of ORco subunits with fluorescent tags in positions that allow FRET/BRET to occur in the case of homomer or heteromer channel pore formation (Machleidt et al., 2015; Scott and Hoppe, 2015; Cranfill et al., 2016) may prove informative. It is also hoped that the application of new approaches to the efforts for structure determination of membrane-anchored receptors including that of single-particle cryo-EM (Baker et al., 2015; Eisenstein, 2016; Tajima et al., 2016) will provide new insights allowing distinction between the two alternatives of our working model and permutations thereof.

Finally, implicit to these findings is the prediction that should natural volatiles with OA-like properties similar to those reported here exist, their use as enhancers of mosquito (and other insect) odor recognition sensitivity could have practical implications. Typical examples include enhanced trapping applications relevant to population surveillance and improved protection measures.

## Author Contributions

PT planned and executed experiments, evaluated results and contributed to the writing of the manuscript; KI planned the project and experimentation, assessed the results and wrote the manuscript.

## Conflict of Interest Statement

The authors declare that the research was conducted in the absence of any commercial or financial relationships that could be construed as a potential conflict of interest.

## Footnotes

1 graphpad.com

2 ch.embnet.org

3 http://topcons.cbr.su.se
