# Supplementary material for: Positive Allosteric Modulation of Insect Olfactory Receptor Function by ORco Agonists
Source: Front Cell Neurosci. 2016 Dec 9;10:275. doi: 10.3389/fncel.2016.00275 (PMC5145856; doi:10.3389/fncel.2016.00275)
Supplement: Supplementary file 1 [file Table_1.PDF]

Supplemental Table 1. Odorants and ORco agonists used in the present study.

Chemical [CAS number]	Abbreviation	Supplier	Purity (%)	Formula
2-Methylphenol (*o*-Cresol) [95-48-7]	2MP	Sigma Aldrich	99+	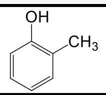
3-Methylphenol (*m*-Cresol) [108-39-4]	3MP	Sigma Aldrich	99	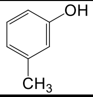
4-Methylphenol (*p*-Cresol) [106-44-5]	4MP	Sigma Aldrich	99	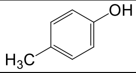
2-Ethylphenol [90-00-6]	2EP	Sigma Aldrich	98.5	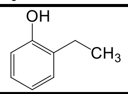
Benzaldehyde [100-52-7]	BA	Sigma Aldrich	>99	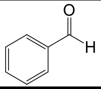
Indole [120-72-9]	IN	Acros Organics	99+	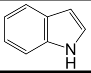
Ethyl butyrate [105-54-4]	EB	Sigma Aldrich	99	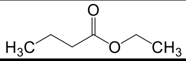
Cyclohexanone [108-94-1]	CH	Sigma Aldrich	≥99.9	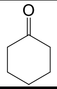
Linalool [78-70-6]	LIN	Sigma Aldrich	95-97	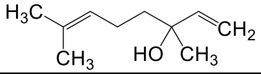
Hexanoic acid [142-62-1]	HEX	Sigma Aldrich	99.5	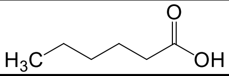
Octanoic acid[124-07-2]	OCT	Sigma Aldrich	99.5	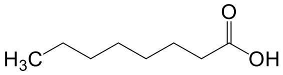
Acetone [67-64-1]	ACE	LabScan Analytical	99.5	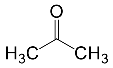
Nonanal [124-19-6]	NON	Sigma Aldrich	97	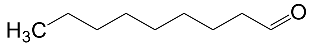
3-Methyl-1-butanol (isoamyl alcohol) [123-51-3]	3MB	Merck	98	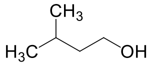
1-Butanol [71-36-3]	BUT	Panreac	99.5	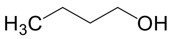
Ethyl acetate [141-78-6]	EA	Merck	99.5	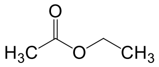
N-(4-ethylphenyl)-2-((4-ethyl-5-(3-pyridinyl)-4H-1,2,4-triazol-3-yl)thio)acetamide [525582-84-7]	VUAA1	Alinda Chemical	>95	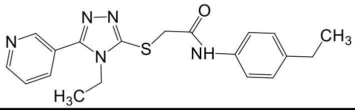
N-(4-ethylphenyl)-2-((4-ethyl-5-(4-pyridinyl)-4H-1,2,4-triazol-3-yl)thio)acetamide [618427-06-8]	OrcoRAM2	Hit2Lead, Vitas-M Lab	>90	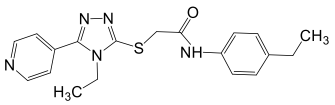
