# Supplementary material for: Positive Allosteric Modulation of Insect Olfactory Receptor Function by ORco Agonists
Source: Front Cell Neurosci. 2016 Dec 9;10:275. doi: 10.3389/fncel.2016.00275 (PMC5145856; doi:10.3389/fncel.2016.00275)
Supplement: Supplementary file 2 [file Table_2.pdf]

Supplemental Table 2. Differences in relative efficacy values obtained with ORcoRAM2. (Upper): Comparison of heteromers and homomer; *relative to ORco. (Middle): Comparison of OA- and SL-induced responses, for heteromers; #relative to 100 µM ORx-specific odorant. (Lower): Synergism between OA and SL; §potentiation relative to 100 µM ORx-specific odorant.

Odorant receptor	Specific odorant (100 µM)	ORcoRAM2 (100 µM)	Relative efficacy*	Figure
ORco	-	+	(1)	1A
OR1/ORco	-	+	3,78	
OR2/ORco	-	+	3.2	
OR9/ORco	-	+	1,05	
OR53/ORco	-	+	3.87	

Odorant receptor	Specific odorant (100 µM)	ORcoRAM2 (100 µM)	Relative efficacy#	Figure
OR1/ORco	+	-	(1)	1C
	-	+	10,22	
OR2/ORco	+	-	(1)	
	-	+	6.22	
OR9/ORco	+	-	(1)	
	-	+	10.4	
OR53/ORco	+	-	(1)	
	-	+	105.26	

Odorant receptor	Specific odorant (100 µM)	ORcoRAM2 (10 µM)	Relative efficacy§	Figure
OR1/ORco	+	-	(1)	3A
	-	+	2.88	
	+	+	32.45	
OR2/ORco	+	-	(1)	
	-	+	0.57	
	+	+	8.09	
OR9/ORco	+	-	(1)	
	-	+	0.05	
	+	+	4.94	
OR53/ORco	+	-	(1)	3B
	-	+	16.3	
	+	+	140.85	
