# Supplementary material for: Positive Allosteric Modulation of Insect Olfactory Receptor Function by ORco Agonists
Source: Front Cell Neurosci. 2016 Dec 9;10:275. doi: 10.3389/fncel.2016.00275 (PMC5145856; doi:10.3389/fncel.2016.00275)
Supplement: Supplementary file 3 [file Data_Sheet_1.pdf]

Supplemental Figure and Table legends

Supplemental Figure S1. Comparison of results obtained with the different assay buffers. Enhanced responses were obtained when modified Ringer solution was used for the assay. «A» stands for standard Ringer, whereas «B» stands for modified Ringer solution. In the experiments presented above (representative experiment for each case), the same cell population was splitted in two, and the experiment was continued identically for two halves, except for the constitution of the Ringer solution used. All agonists added (IN, 2EP, OA=ORcoRAM2) were at 100 μM. Similar results were obtained with remaining heteromers, as well as for heteromers with the ORco agonist (not shown).

Supplemental Figure S2. Representative results obtained using VUAA1 as ORco agonist. (A). Direct agonism of OR1/ORco with OA and 4MP : representative result from an experiment performed in duplicates. VUAA1 was at 50 μM, 4MP at 100 μM. **(B).** Potentiation of OR1/ORco: mean of two experiments, each performed in duplicates. Odorants (3MP and 4MP) were added at 100 μM, and VUAA1 at 10 μM. **(C).** Potentiation of responses of OR9/ORco to 2EP by both ORco agonists used, namely VUAA1 (OA1) and ORcoRAM2 (OA2). In this case OAs were added at 50 μM, while 2EP at 100 μM.

Supplemental Figure S3. The isoforms of the olfactory receptor subunits OR9 and OR53 used in this study. (A). The OR9 variant. The top line indicates the gene structure with Exons (I) and Introns (I) numbered consecutively and their lengths indicated in parentheses. The second line presents details of the splicing at the E4/I4/E5 junction sequences (***Seq1***) listed in VectorBase (ID AGAP008333-RA) with the corresponding amino acids indicated below the nucleotide sequence. The third line presents the sequences of the alternatively spliced form (***Seq2***) used in this study, with the additional nucleotides (and corresponding amino acids) contributing to the alternative E5' indicated in bold. **(B).** *Left:* The alignment of the amino acid sequences of the originally reported (***Seq1***) and the alternatively spliced variant of OR9 (***Seq2***) flanking the E4/E5 (E4/E5' for ***Seq2***) splice junction; *Right:* the insertion of the 9 amino acids (black box) occurs in the presumed second intracellular loop (*ICL2*) of OR9. **(C).** The OR53 variant. The top and middle schematics show the previously reported (VectorBase ID AGAP009390-RA) gene structure and spliced sequences of OR53 at the E1/I1/E2 junctions (***Seq1***) with the third line showing the alternative splicing event at the E1'/I1' junction that gave rise to the OR53 variant used in this study, which differed from the previously published isoform by an extension of the first exon,

translated into the incorporation of the additional 8 amino acids and the D to E conversion in position #161 (***Seq2***). (D). *Left:* The alignment of the amino acid sequences of the originally reported (***Seq1***) and the alternatively spliced variant of OR53 (***Seq2***); *Right:* the insertion of the 8 amino acids (black box) occurs in the presumed second extracellular loop (*ECL2*) of OR53. Additional single nucleotide polymorphisms in both sequences are underlined.

Supplemental Figure S4: Functional expression of ORs 9 and 53 - odorant recognition. (**A)** OR9 was expressed together with ORco and Photina, and responses were measured after challenging the cells with the indicated odorants at 100 μM concentration (left diagram, n=4 for 2EP, 4MP; 3 for EB and 3MP; and 2 for CH and IN). The dose-response curve for OR9 with 2EP is presented at right (n=2) based on same experimental results as those presented in figure 4A in the absence of OA. **(B)** OR53/ORco expressing cells were tested with eleven odorants at 100 μM, and responses were normalized to the response to linalool (left, n=2 except for hexanoic acid and 1-butanol, where results are from one experiment performed in triplicates). As shown by the inset at right, the responses to linalool were very small, but this was not due to lack of general responsiveness of these cells, as shown after challenging them with Triton X-100, which allows Ca^{2+} entry into them (n=2). Results from an analogous experiment with OR2/ORco are shown for comparison in the right inset.

Supplemental Figure S5. Comparison of heteromer responses for direct agonism by odorants and ORco agonists, at concentrations that are more close to equipotency (between the two agonists, for each receptor). Magnitudes of responses are shown for OR1/ORco with 100 μM OA and 10 μM 4MP, and for OR53/ORco with 100 μM OA and 1 mM LIN, all added independently. OA used was ORcoRAM2. Representative results for two of the heteromers, to further show that our conclusion regarding the vast differences in the efficacy of OA-agonism of heteromers vs. odorant-agonism is valid (**Fig. 1C**), despite the fact that it was shown by using 100 μM of both and not equipotent concentrations. Representative experiments perfomed in triplicates are shown.

Supplemental Figure S6. Heteromeric channel responses at equipotent concentrations of odorants (EC_{20} values) and potentiation by 10 μM OA (ORcoRAM2). (A). Results as replotted from Fig 4. Concentration of the odorant at EC_{20}: 4MP (for OR1) 427nM, IN (for OR2) 3.3 μM, 2EP (OR9) 35 μM and LIN (OR53) 105 μM. **(B).** Comparative potentiation magnitudes observed at EC_{20} of odorants (from diagrams presented above) are as follows: OR1 ~93, OR2 ~53, OR9 ~10 and OR53 ~253 fold. Remark: No standard deviations appear,

because data for the specific concentration at EC_{20} come from the fitted line in the dose-response curves.

Supplemental Table 1. Odorants and ORco agonists used in the present study. CAS numbers are in brackets and common names in parentheses. Information is also provided for the corresponding abbreviations used in the figures, the suppliers and purity of the chemicals used in this study as well as their structural formulae.

Supplemental Table 2. Differences in relative efficacy values obtained with ORcoRAM2. (Upper): Comparison of heteromers and homomer; *relative to ORco. (Middle): Comparison of OA- and SL-induced responses, for heteromers; #relative to 100 µM ORx-specific odorant. (Lower): Synergism between OA and SL; §potentiation relative to 100 µM ORx-specific odorant.
