# Supplementary figures and images for: Positive Allosteric Modulation of Insect Olfactory Receptor Function by ORco Agonists
Source: Front Cell Neurosci. 2016 Dec 9;10:275. doi: 10.3389/fncel.2016.00275 (PMC5145856; doi:10.3389/fncel.2016.00275)

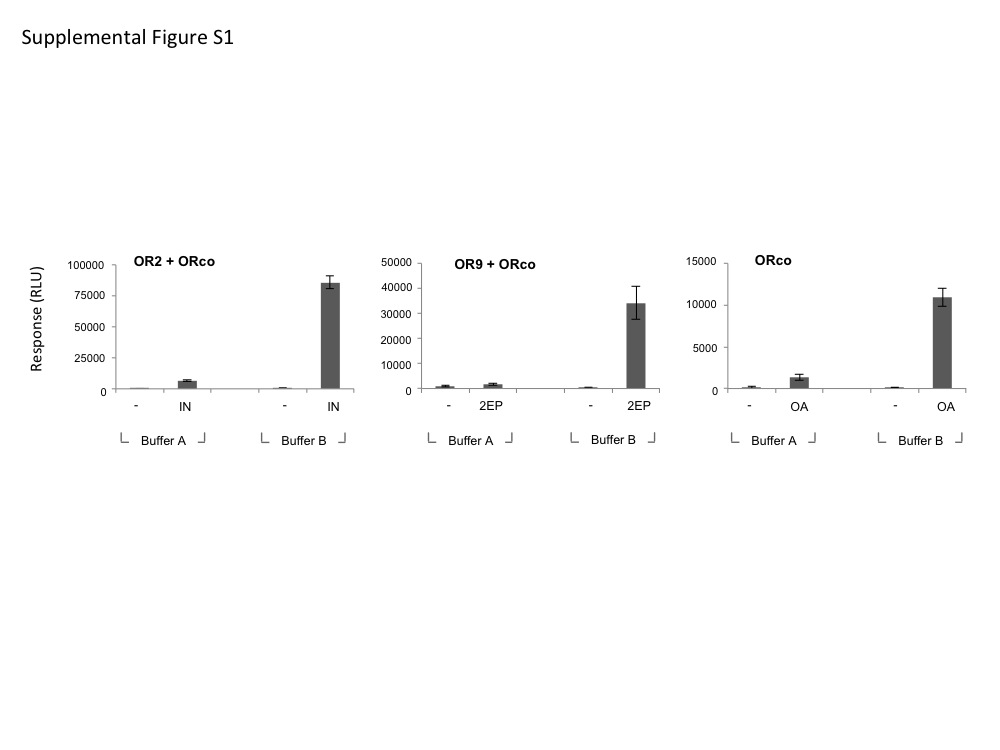

Supplement: Supplementary file 4 [file Image_1.JPEG]

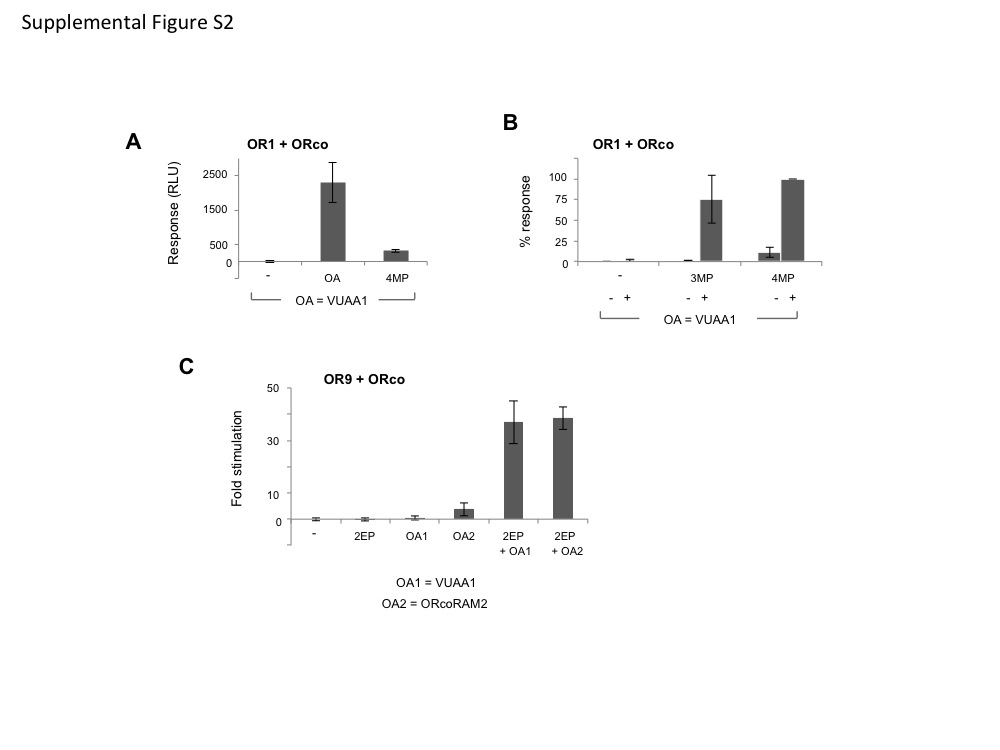

Supplement: Supplementary file 5 [file Image_2.JPEG]

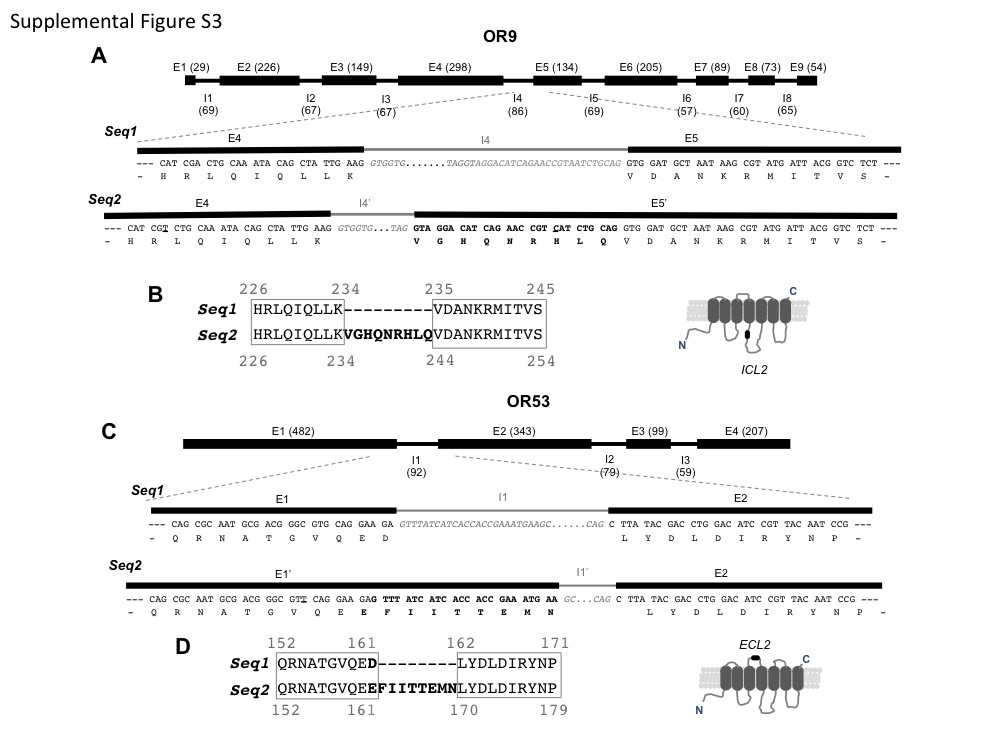

Supplement: Supplementary file 6 [file Image_3.JPEG]

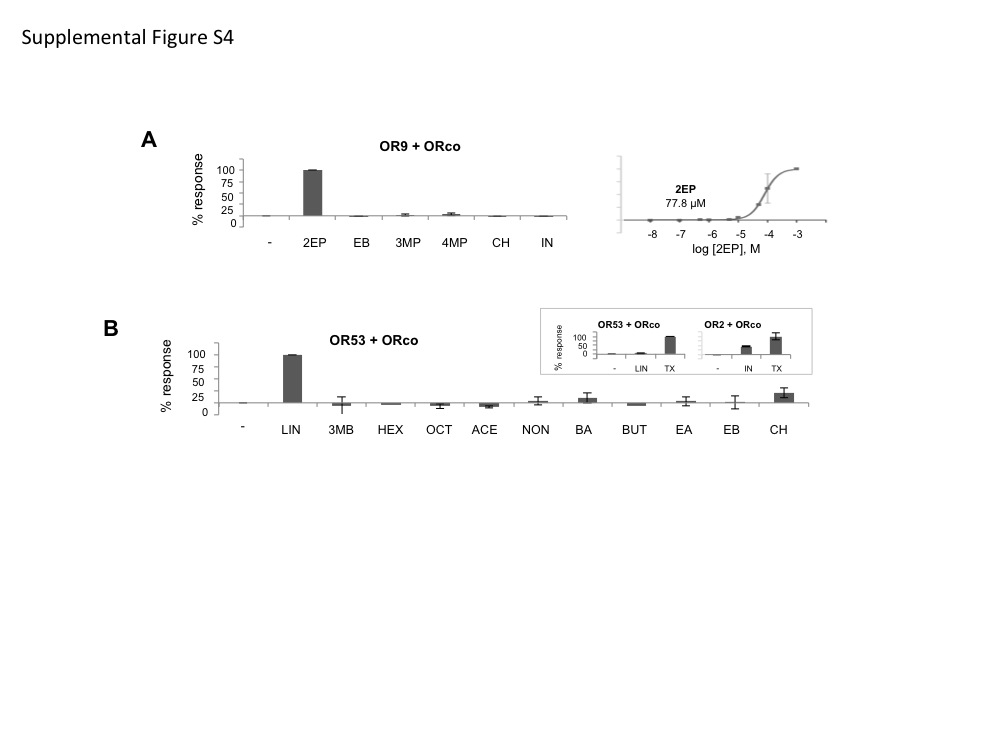

Supplement: Supplementary file 7 [file Image_4.JPEG]

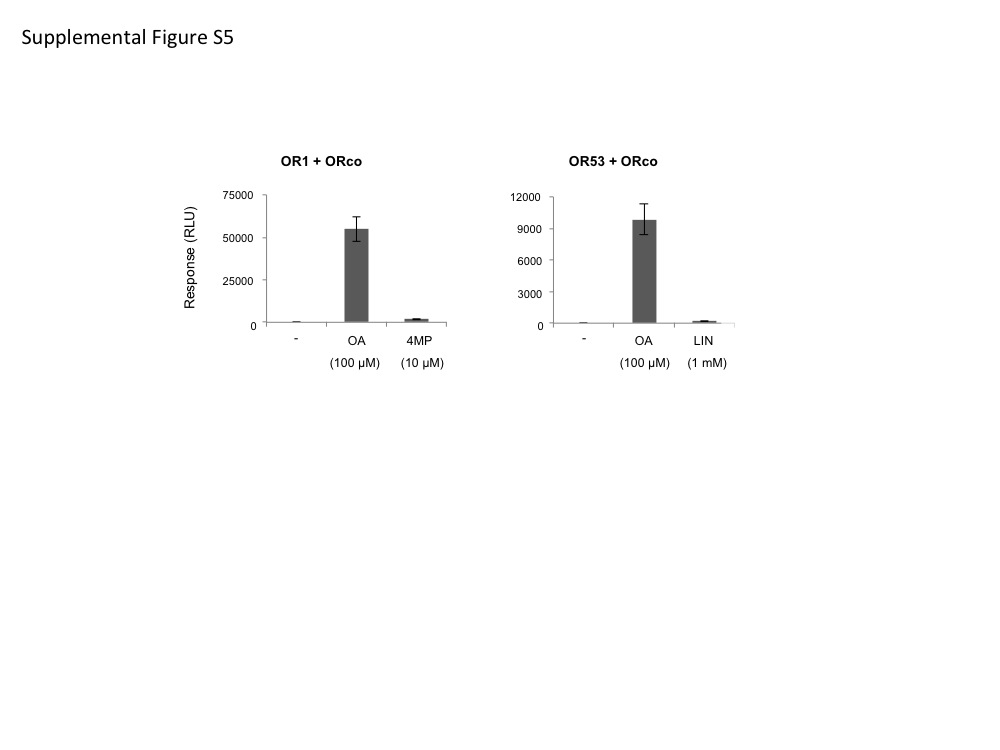

Supplement: Supplementary file 8 [file Image_5.JPEG]

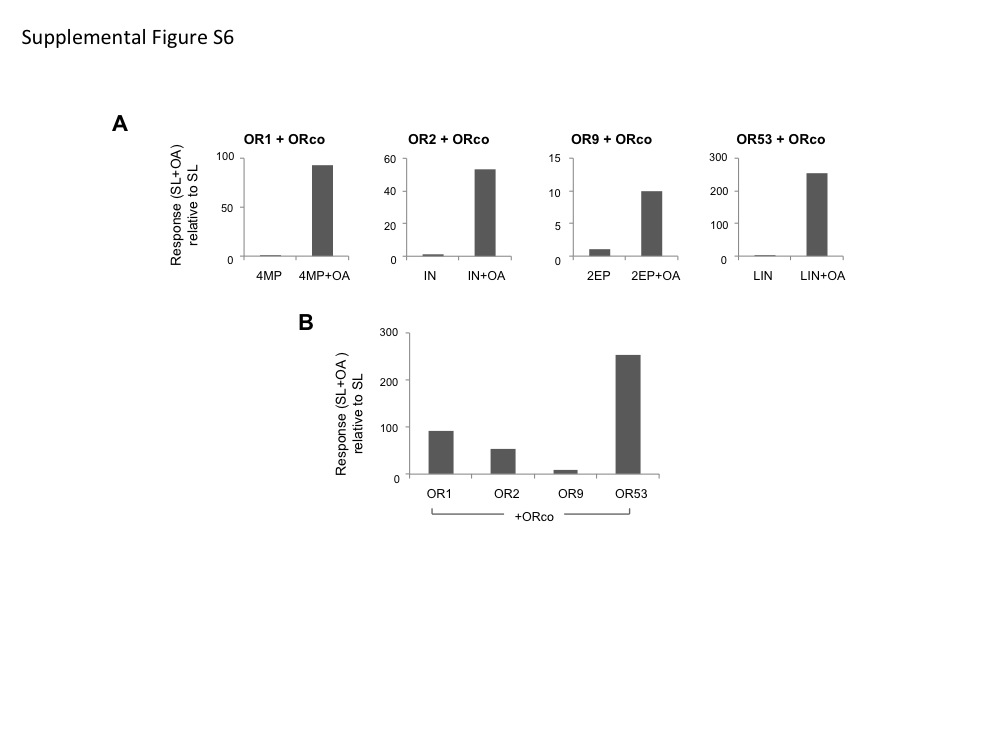

Supplement: Supplementary file 9 [file Image_6.JPEG]
